# Emission engineering in microdisk lasers via direct integration of meta-micromirrors

**DOI:** 10.1515/nanoph-2023-0898

**Published:** 2024-04-17

**Authors:** Aran Yu, Moohyuk Kim, Da In Song, Byoung Jun Park, Hae Rin Jeong, Byeong Uk You, Seung-Woo Jeon, Sang-Wook Han, Myung-Ki Kim

**Affiliations:** KU‐KIST Graduate School of Converging Science and Technology, 34973 Korea University, Seoul, 02841, Republic of Korea; Mechatronics Research Center, SAMSUNG Electronics, Hwaseong 18448, Republic of Korea; Center for Quantum Information, 58975 Korea Institute of Science and Technology (KIST), Seoul, 02792, Republic of Korea; Division of Nanoscience and Technology, KIST School, 34973 Korea University of Science and Technology (UST), Seoul, 02792, Republic of Korea; Department of Integrative Energy Engineering, 34973 Korea University, Seoul 02841, Republic of Korea

**Keywords:** small lasers, microdisk lasers, metasurfaces, meta-micromirrors

## Abstract

Despite their excellent performance and versatility, the efficient integration of small lasers with other optical devices has long been hindered by their broad emission divergence. In this study, we introduce a novel approach for emission engineering in microdisk lasers, significantly enhancing their vertical emission output by directly integrating specially designed reflective metalenses, referred to as “meta-micromirrors”. A 5 μm-diameter microdisk laser is precisely positioned at an 8 μm focal distance on a 30 × 30 μm^2^ meta-micromirror featuring a numerical aperture (NA) of 0.95, accomplished through micro-transfer printing techniques. Our experiments demonstrated a notable increase in the emission efficiency within an NA of 0.65. Specifically, we observed a 2.68-fold increase in the average emission from ten microdisk lasers. This integration not only enhances the emission efficiency of small lasers but also holds considerable implications for micro- and nano-photonic integrations. The results of this integration open up new possibilities in various fields, including photonic integrated circuits, bio-sensing technologies, and the development of quantum light sources.

## 1 Introduction

In today’s rapidly evolving technological landscape, the demand for smaller, more efficient, and versatile devices has intensified. As a result, smaller lasers, such as micro- and nano-lasers, have attracted considerable interest. These lasers play vital roles across diverse sectors, including optical communications [1], [2], [3], [4], [5], [6], [7], [8], optical sensing [9], [10], [11], [12], [13], [14], defense and security [15], [16], and biomedical applications [17], [18], owing to their compact sizes and superior performance capabilities. Their integration into various technological domains not only enhances existing applications, but also paves the way for novel innovations, profoundly shaping the future trajectory of multiple fields. Nevertheless, the small size of these lasers presents a critical drawback: their wide emission angle. This characteristic causes laser light to disperse over a broader area, severely impeding seamless integration with other technologies [19], [20], [21], [22], [23]. This wide emission angle complicates the coupling with external optical devices, posing challenges in device design and fabrication. At times, it necessitates additional optical adjustments or complex alignment procedures.

Recently, light manipulation using metastructures, such as metamaterials and metasurfaces, has garnered significant attention [24], [25], [26], [27], [28]. Metasurfaces, comprising nanostructure arrays designed to manipulate electromagnetic fields with a small thickness, offer precise control over light properties, including phase, direction, amplitude, and polarization [29], [30], [31], [32]. Their thin, lightweight nature, and versatility enable functionalities like high-refractive-index materials [33], [34], [35], negative refraction [36], [37], [38], [39], and high-numerical-aperture (NA) metalenses [40], [41], [42], [43], [44]. In recent years, the integration of ultrasmall light sources, such as diamond nitrogen-vacancy (NV) centers and quantum dots, with low-loss, high-efficiency dielectric metasurfaces has been actively studied [45], [46], [47], [48], [49], [50]. However, the integration of metasurfaces and small lasers remains a significant challenge because it requires precise alignment and effective coupling within a limited small form factor.

In this study, we present a novel approach to enhance the emission efficiency of microdisk lasers (which are small lasers known for their wide emission angles) by directly integrating them with reflective metalenses, named “meta-micromirrors”. Employing micro-transfer printing techniques, we positioned the 5 μm-diameter microdisk laser at an 8 μm focal distance on a 30 × 30 μm^2^ meta-micromirror featuring an NA of 0.95. Our experiments demonstrated a notable increase in the emission efficiency within an NA of 0.65, evidenced by a 2.68-fold increase in average emission observed from ten microdisk lasers. This integration not only enhances the emission efficiency of small lasers, but also has far-reaching implications for future micro- and nano-photonic integrations, heralding new possibilities in fields such as optical communications and biological applications.

## 2 Device concept

**Figure 1:**
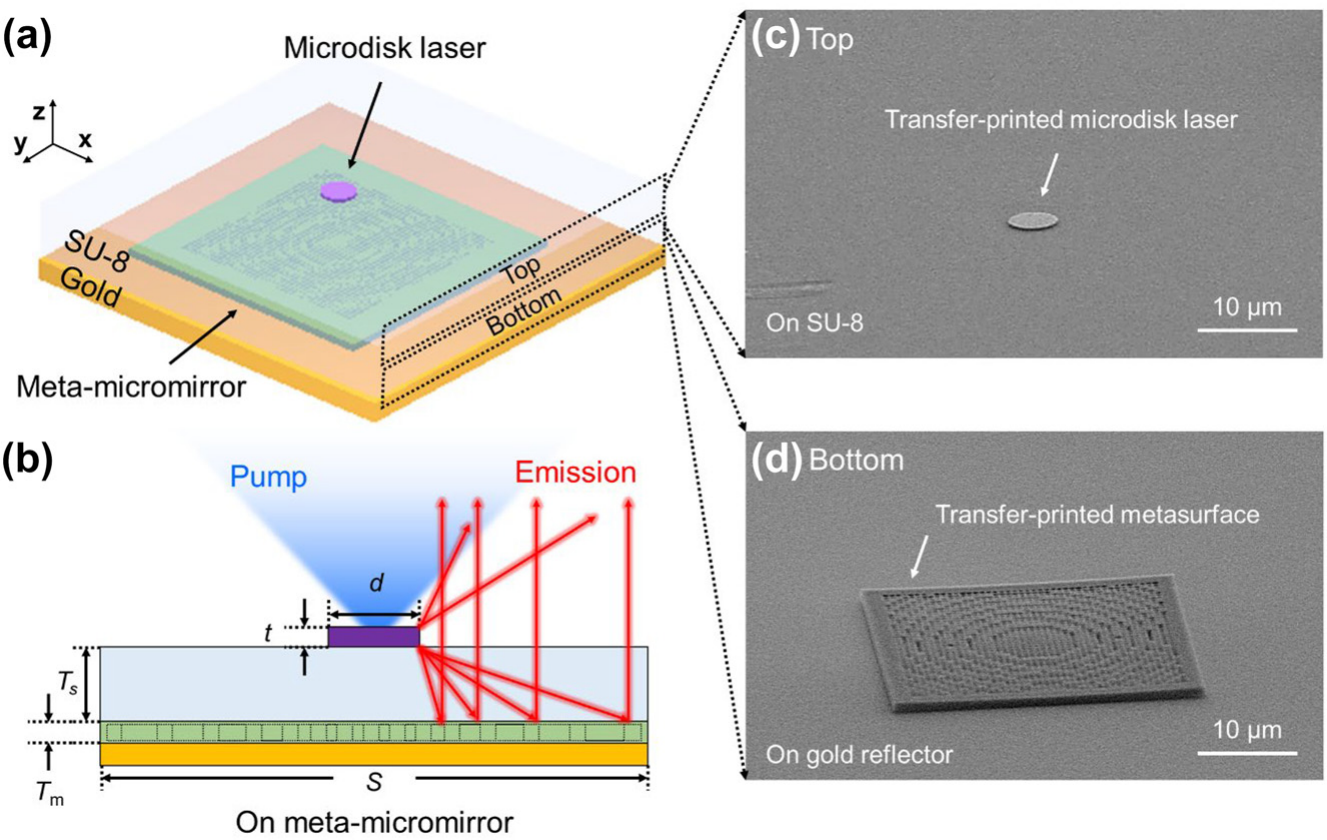
Microdisk laser integrated with meta-micromirror. (a) Schematic of microdisk laser integrated with meta-micromirror. The meta-micromirror is formed by placing a SiN metasurface on top of a gold reflector, which is separated from the microdisk laser by an SU-8 spacer. (b) Beam path of the microlaser in the presence of the meta-micromirror. Here, the thickness of the SU-8 layer (*T*_*s*_) is adjusted to match to the focal length (*f*) of the meta-micromirror. (c, d) Scanning electron microscopy (SEM) images of the microdisk laser and metasurface, transfer-printed on SU-8 and a gold reflector, respectively.

Figure 1(a) presents a schematic diagram of the microdisk laser integrated with a micromirror. This configuration addresses the inherent challenge of whispering-galley-mode microdisk lasers, which struggle with vertical laser-beam collection because of their substantial emission angles. This design introduced a reflective mechanism at the base that merged the laser beams emitted in two vertical directions into a singular vertical trajectory. However, given the inherently wide emission angle of a microdisk laser, a unidirectional reflector alone is insufficient to optimize the vertical emission efficiency. Therefore, it is essential to design a lower reflector that not only reflects, but also strategically redirects the beam’s emission path to enhance its performance. To achieve this redirection, a highly specialized ultrathin reflective metalens with concave mirror capabilities, called a meta-micromirror, is strategically positioned beneath the microdisk laser. This placement allowed the steering of the high-angle laser beams emitted downward to be redirected upward, as shown in Figure 1(b). Here, an SU-8 spacing layer was employed to maintain the gap between the meta-micromirror and the microdisk laser precisely.

Optimizing the performance involves meticulously adjusting the SU-8 layer thickness (*T*_s_), size (*S*), and focal length (*f*) of the meta-micromirror structure, considering the laser’s radiation angle. The laser mode selection is dependent on the dimensions of the disc (diameter = *d*, thickness = *t*), whereas the efficacy of the meta-structure is adjusted through the structure’s thickness (*T*_m_) and the arrangement of the introduced nano-air holes. The microdisk laser is based on indium–gallium–arsenide–phosphide (InGaAsP) with embedded quantum wells to allow emission in the communication wavelength spectrum. The meta-micromirror is designed on a silicon nitride (SiN) base, offering a thickness of ∼1 μm in the communication wavelength with high efficiency. The microdisk laser and meta-micromirror are, respectively, transfer-printed onto an SU-8 layer and a gold (Au)-reflector, as shown in Figure 1(c) and (d). This process facilitated the fabrication of a compact and highly efficient laser platform that could be seamlessly integrated with a meta-micromirror.

## 3 Device design

Our design approach for the meta-micromirror prioritized both thinness and high efficiency, aiming for seamless integration with small laser systems. For the metastructure material, we chose SiN because of its high transmittance in the communication wavelength range and well-balanced refractive index. These attributes enable it to induce full phase shifts in layers as thin as ∼1 μm, aligning well with our design objectives. While silicon (Si) offers higher refractive-index values, it leads to significant surface reflections, diminishing the overall efficiency of the meta-structure. In contrast, SiNs can be precisely nanofabricated and are highly resistant to changes in temperature and humidity. To achieve the desired phase modulation, nanosized air holes were introduced into the SiN layer. These air holes, with dimensions significantly smaller than the operational wavelength, allowed us to modify the effective refractive index of the material, thereby facilitating adjustable phase variations. Although SiN rod structures could also be viable for similar phase shifts, we found that a mesh-type air-hole pattern was more beneficial, especially for transfer-printing processes.

**Figure 2:**
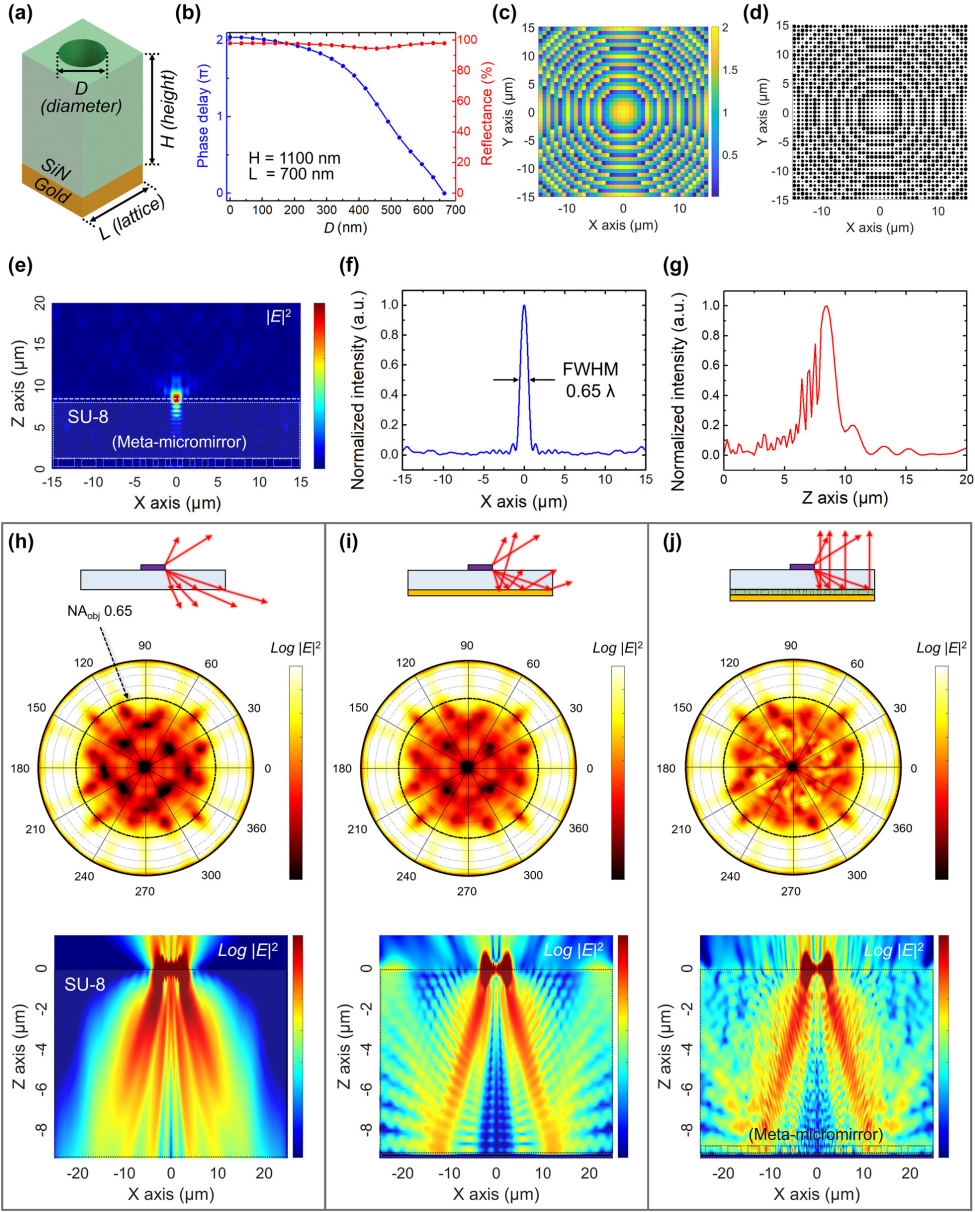
Design of meta-micromirror. (a) Schematic of unit cell of meta-micromirror. The unit cell with size *L* consists of a gold reflector, SiN of height *H*, and an air hole with diameter *D*. (b) Calculated phase delay as *D* varies from 35 nm to 665 nm at a wavelength of 1550 nm, *H* = 1100 nm, and *L* = 700 nm. (c) Phase map of a focal length of 8 μm in an external environment of SU-8 (refractive index = 1.61). (d) A map of *D* of meta-micromirrors corresponding to a phase map with a focal length of 8 μm. The total area here is 30 × 30 μm^2^, where the numerical aperture (NA) is 0.95. (e) Distribution of the calculated electric field intensity (|*E*|^2^) on the *xz*-plane, illustrating scenario of a planewave incident from above, travelling along the negative *z*-direction. (f) |*E*|^2^ distribution along the *x*-direction. The full-width at half-maximum (FWHM) of the focused beam is measured to be 1.02 μm, which is about 0.65 times the wavelength. (g) |*E*|^2^ distribution along the *z*-direction. The depth of focus (DoF) was measured to be 743 nm. (h–j) Saturated far-field distributions and logarithmic scale of |*E*|^2^ on the *xz*-plane for scenarios where a microdisk laser is placed on an SU-8 layer without any reflector, on an SU-8 layer equipped solely with a gold reflector, and on an SU-8 layer that includes a meta-micromirror. In these simulations, the microdisk laser has a diameter (*d*) of 5 µm and a thickness (*t*) of 220 nm, and emits a beam with *λ* of 1556.23 nm with an azimuthal mode number of 24.

Figure 2(a) illustrates the unit cell of a meta-micromirror, commonly referred to as a meta-atom. The SiN thickness (*T*_m_) and unit cell size (*L*) were set to the optimal values of 1100 and 700 nm, respectively [see Supplementary Note 1]. Figure 2(b) showcases the calculated phase delay in the reflected beams as the air hole diameter (*D*) varies. The calculations were conducted using a finite-difference time-domain (FDTD) simulation provided by Lumerical Inc. This figure clearly demonstrated the achievement of a 2π phase shift across *D* range from 35 to 665 nm, while consistently maintaining an average reflectance of 97 %.

Based on the data in Figure 2(b), we constructed the phase map of a concave meta-mirror using the following equation:

(1) $$\Phi \left(x,y\right)=\frac{2\pi }{\lambda /n_{b}}\left(\sqrt{\left(x^{2}+y^{2}\right)+f^{2}}-f\right)$$

Here, *λ*, *n*_*b*_, *f* represent the operating wavelength, the refractive index of the background, and the focal length of the meta-micromirror, respectively. Figure 2(c) depicts the calculated phase map (Φ) of a meta-micromirror, featuring a focal length (*f*) of 8 μm, in conditions with an operating wavelength (*λ*) of 1550 nm and a background of SU-8 with *n*_*b*_ of 1.61. Subsequently, we constructed the structure of the meta-micromirror, as presented in Figure 2(d), by mapping *D* according to the phase map shown in Figure 2(c). For the actual fabrication process, the meta-micromirror was designed as a square with each side (*S*) measuring 30 μm, resulting in an NA (that is, NA_meta_) of 0.95.

To evaluate the performance of the meta-micromirror, we conducted FDTD simulations utilizing the structure depicted in Figure 2(d). Figure 2(e) illustrates the distribution of the calculated electric field intensity (|*E*|^2^) on the *xz*-plane, representing the scenario of a plane wave incident from above, propagating along the negative *z*-direction. This figure distinctively showcases that the reflected beam is focused ∼8 μm above the top of the meta-structure, closely aligning with the targeted design value of 8.0 μm. With this configuration, the full-width at half-maximum (FWHM) of the focused beam was measured to be 1.02 μm, which is about 0.65 times the wavelength, as illustrated in Figure 2(f). Additionally, the measured depth of focus (DoF) was 743 nm, as shown in Figure 2(g). This figure also reveals the interference pattern within the SU-8 layer, which is attributed to Fabry–Perot interference caused by reflections from both the top and bottom surfaces of SU-8. The reflectance efficiency of the beam concentrated near the focus was determined to be 60.3 %.

In the configuration shown in Figure 2(e), placing a microdisk laser above the focused area of the SU-8 layer implies that the laser beam travelling downward is efficiently reflected within the operating range of the meta-micromirror. Consequently, this redirection aligns the beam towards a nearly vertical trajectory. In this context, the FWHM in Figure 2(f) represents the alignment tolerance of the microdisk laser, and the DoF in Figure 2(g) can be interpreted as the permissible thickness variation of the laser. Figure 2(h)–(j) display the far-field distribution and logarithmic scale of |*E*|^2^ on the *xz*-plane under different scenarios: placing a microdisk laser on an SU-8 layer without any reflector, on an SU-8 layer equipped solely with an Au-reflector, and on an SU-8 layer that includes a meta-micromirror, respectively. In these simulations, the microdisk laser has a diameter (*d*) of 5 µm and a thickness (*t*) of 220 nm, emitting a beam with *λ* of 1556.23 nm with an azimuthal mode number of 24. As shown in Figure 2(h), 42 % of the total energy emitted from the microdisk laser was projected upward into the air. Of the energy emitted upwards, only 2.5 % was detected within NA = 0.65 (corresponding emission angle <40.5°). In the configuration presented in Figure 2(i), which employs only an Au-reflector, the predominant portion of the upwardly reflected beam is characterized by wider angles. This enhances the efficiency of the beam entering within NA = 0.65, showcasing a modest increase of 1.05 times in comparison with the scenario depicted in Figure 2(h). However, in a structure that integrates a meta-micromirror, as shown in Figure 2(j), the upward-reflected beam is steered and emitted in the vertical direction. This behavior is observable in the far-field pattern and |*E*|^2^ profile. In this structure, the efficiency of the beam emitted within an NA of 0.65 is calculated to increase by 1.39 times compared with the scenario in Figure 2(h). It is also confirmed that the enhancement in efficiency is not attributed to scattering by the meta-structure, but rather to the meticulously engineered phase distribution, as detailed in Supplementary Note 2. In addition, the change in enhancement due to shifts in resonance wavelength, or more specifically, the displacements in disk diameter of ±2 μm, does not significantly deteriorate [Supplementary Note 3]. Notably, this efficiency enhancement becomes more significant as the NA of the collecting objective lens (NA_obj_) decreases. For example, the efficiency enhancement increases by a factor of 4.2 when NA_obj_ is decreased to 0.5, peaking at a factor of 8.5 when NA_obj_ is further reduced to 0.39 [see Supplementary Note 4]. The simulations revealed that the integrating a meta-micromirror substantially improved the unidirectional vertical emission of the microdisk laser, achieved through an emission engineering process.

## 4 Fabrication

**Figure 3:**
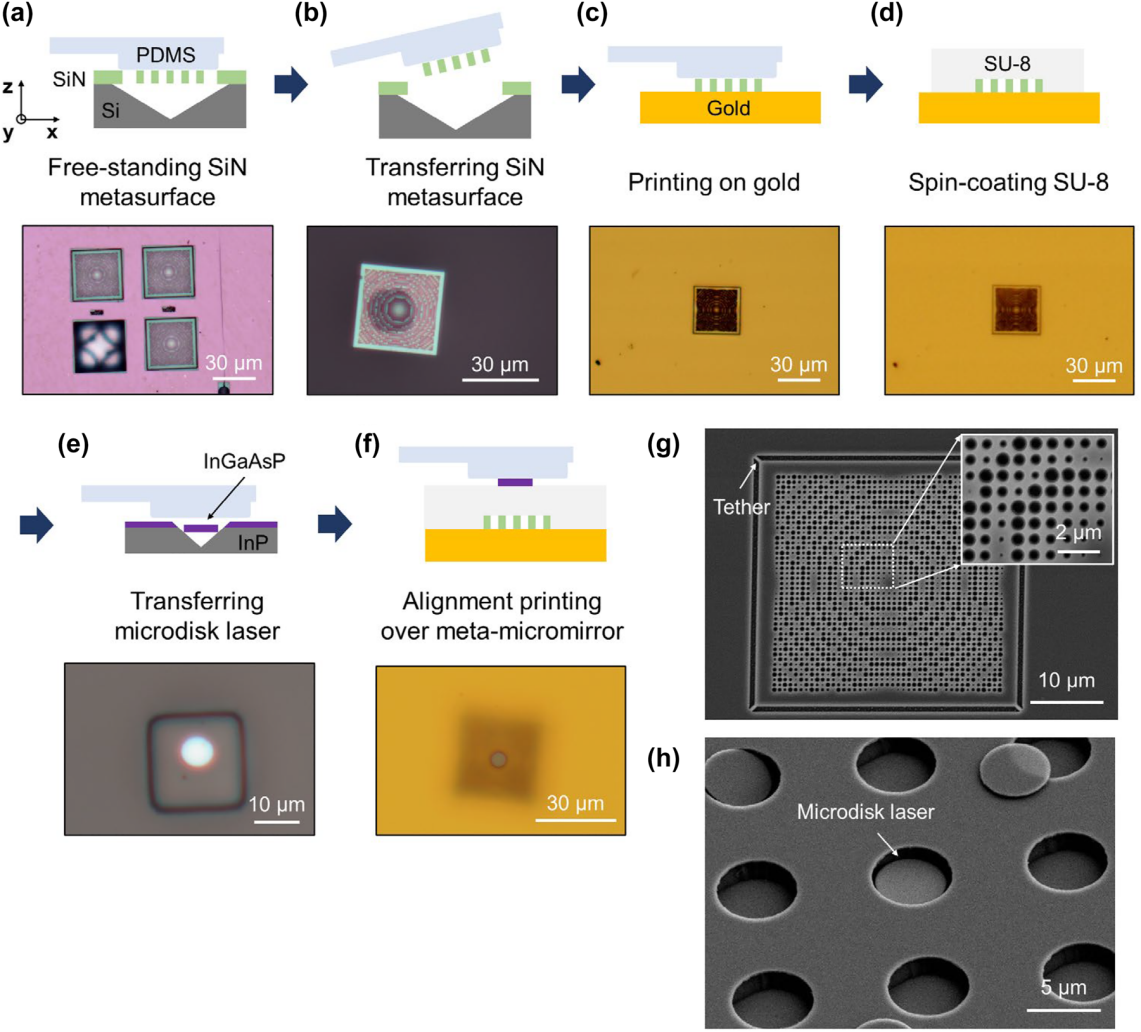
Fabrication process. (a–c) Transfer printing process of free-standing metastructure onto gold reflector. For transfer printing, a 20 × 20 μm^2^ polydimethylsiloxane (PDMS) stamp was used to peel the metastructure from the SiN thin film and print it onto the gold reflector while observing it under a optical microscope. (d) SU-8 spin-coating process to form an 8 μm-thick spacer on top of the meta-micromirror. (e–f) Alignment transfer-printing process of InGaAsP microdisk laser onto SU-8/meta-micromirror using PDMS stamp. (g) SEM image of free-standing SiN metasurface with four tethers. (h) SEM image of InGaAsP microdisk laser after released from the wafer.

To fabricate the microdisk laser integrated with a meta-micromirror, as depicted in Figure 3, we created a SiN-based metastructure, transferred it onto an Au-reflector by transfer-printing, and carefully positioned the prefabricated InGaAsP microdisk laser on top of the meta-micromirror separated by the SU-8 spacer. Initially, to create a transferable SiN metastructure, we deposited 1.1 μm-thick SiN on a Si wafer, and then patterned the metastructure using electron beam (e-beam) lithography, followed by dry etching with reactive ion etching (RIE) process to form the SiN-based metastructure layer. This was followed by potassium hydroxide (KOH) wet-etching to create a transferable freestanding SiN metastructure layer [see Supplementary Note 5]. Afterwards, as shown in Figure 3(a–c), the free-standing metastructure was transfer-printed onto an Au-reflector. For this, a 20 × 20 μm^2^ polydimethylsiloxane (PDMS) stamp was used to peel off the metastructure from the SiN thin film, and it was printed onto the Au-reflector while observing with an optical microscope. As shown in Figure 3(g), tether structures, optimized to dimensions of 2 μm in length and 500 nm in width, were introduced at the edges of the SiN metastructure. The structures were separated from the SiN film by applying mechanical pressure employing a PDMS stamp. To ensure secure attachment of the SiN metastructure to the Au-reflector, the Au-surface underwent preparatory treatment involving a 50-W oxygen gas (O_2_) plasma exposure for 10 min. Subsequently, as seen in Figure 3(d), SU-8 was spin-coated at 2000 rpm for 30 s, followed by post-baking at 95 °C for 6 min, to form an ∼8 μm-thick spacer over the meta-micromirror. Following this, the coated SU-8 was treated again with 50 W of O_2_ plasma for 10 min for effective printing using the InGaAsP laser.

Independently of the SiN metastructure fabrication, we manufactured microdisk lasers on InGaAsP/InP wafers with a diameter of 5 μm and a thickness of 220 nm [see Supplementary Note 6]. After patterning with e-beam lithography, we employed chemically-assisted ion-beam etching (CAIBE) for dry etching, followed by wet etching with hydrochloric acid (HCl) to separate the InGaAsP microdisk lasers from the InP substrate. The microdisks, which were designed without tethers, had freedom of movement. To facilitate transfer-printing, we strategically patterned the edges such that they were exceptionally thin. This design choice allowed the microdisks to remain delicately but securely attached near the edges, enabling effective transfer while preserving their structural integrity, as illustrated in Figure 3(h). Subsequently, as depicted in Figure 3(e) and (f), we used a PDMS stamp to transfer-print the InGaAsP microdisk lasers onto the fabricated SU-8 layers. In this process, alignment precision is critical, necessitating accurate alignment between the center of the meta-micromirror and the microdisk laser. To accomplish this, we employed high-precision nanostages with a high-resolution microscope system, achieving a minimal alignment deviation of below 500 nm, which is smaller than the size of the individual unit cells. For a comparative analysis of the performance of microdisk lasers integrated with meta-micromirrors, we prepared two sets of microdisk laser samples: ten samples integrated with meta-micromirrors and another three samples paired exclusively with Au-reflectors. This approach enabled us to directly compare the performance outcomes of the microdisk lasers in the context of their integration with either meta-micromirrors or AU-reflectors.

## 5 Measurement

**Figure 4:**
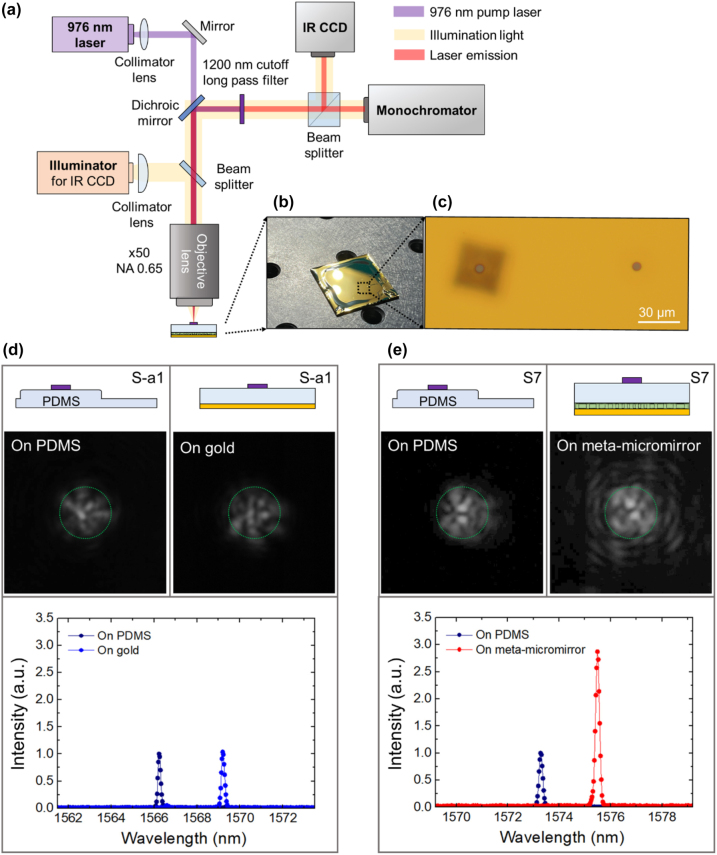
Characterization of microdisk laser emission. (a) Photoluminescent setup with a 976-nm pump laser. The images and intensities of the laser output beams are detected using an infrared charge-coupled device (IR CCD), following the removal of the pumping beam through a dichroic mirror and a long-pass filter. The laser spectrum is analyzed using a spectrometer. Here, a ×50 IR objective lens with a NA_obj_ of 0.65 is used to collect the laser emission. (b) Photograph of the sample containing both the meta-micromirror and the microdisk laser. (c) Microscope image of two configurations, one with a microdisk laser mounted on a meta-micromirror and the other with a microdisk laser positioned on a gold reflector. (d) IR CCD image and emission spectrum of a microdisk laser printed on a gold reflector without meta-structure, compared to results from the same laser mounted on PDMS. The green dotted circle provides a guideline for comparing sizes. (e) IR CCD image and emission spectrum of a microdisk laser printed on a meta-micromirror, compared to results from the same laser mounted on PDMS.

Before validating the performance of the microdisk laser integrated with the meta-micromirror, we evaluated its effectiveness using a reflective imaging setup. As detailed in Supplementary Note 7, we found that the fabricated meta-micromirror effectively focused the beam ∼8 μm above the top surface of the meta-structure, i.e., on the upper surface of the SU-8 layer, matching our initial design specification. After printing the microdisk laser onto the verified meta-micromirror, we tested its laser performance using the photoluminescent setup shown in Figure 4(a). In this arrangement, we employed a 976 nm wavelength laser as the pumping source. To ensure the stable operation of the microdisk laser, we applied pulsed pumping (repetition rate = 1 MHz, pulse width = 50 ns). To maintain experimental consistency, we kept the pumping conditions uniform across all samples, as depicted in Figure 1(b) [Supplementary Note 8]. The image and intensity of the emitted laser output beam were detected using an infrared charge-coupled device (IR CCD) following the removal of the pumping beam through a dichroic mirror and a long-pass filter. The laser spectra were analyzed using a spectrometer. For this setup, a ×50 IR objective lens with an NA_obj_ of 0.65 was utilized to collect the emitted microdisk laser beam. Figure 4(b) showcases a photograph of the sample containing both the meta-micromirror and microdisk laser after all the fabrication processes were completed. The microscope image in Figure 4(c) illustrates two configurations prepared for comparison: one featuring the microdisk laser mounted on the meta-micromirror, and the other with the microdisk laser positioned on an Au-reflector.

Before printing the microdisk lasers when they were positioned solely on the PDMS stamp, we measured the intensities, images, and spectra of the microdisk laser emissions for comparison, as shown in Figure 4(d) and (e). This was performed prior to measuring the emission characteristics of the same laser on both an Au-reflector and a meta-micromirror. The refractive index of PDMS (*n*_PMDS_ = 1.40) differs by ∼0.21 from that of SU-8 (*n*_SU-8_ = 1.61), and this variance was found to be negligible in the context of the beam emission conditions. Consequently, a laser on PDMS was employed as a reference standard for each set of measurements. Note that, in the transfer printing process utilizing PDMS stamps, only negligible amounts of PDMS residue are left on the laser [Supplementary Note 9]. Figure 4(d) presents the IRCCD images and emission spectrum results for a microdisk laser printed on an Au-reflector without a metastructure, compared with the results from the same laser mounted on PDMS. For the structure placed on PDMS, the laser wavelength was measured at 1566.25 nm, and its threshold peak power was measured at 674 µW [see Supplementary Note 10]. On the other hand, the same laser positioned on the Au-reflector underwent a redshift to a wavelength of 1569.21 nm, which was attributed to the relatively higher refractive index of the SU-8 spacer compared to that of PDMS. The threshold power for this laser was measured at 772 μW. The total power enhancement collected from the laser on the Au-reflector in comparison with that on PDMS was observed to be ∼1.04 times, exhibiting a very slight change. Furthermore, the emission images reveal a slightly increased size relative to the reference images, indicating a slight expansion in the directional range of the beam, which was not previously detected. In the case of the microdisk laser located on the meta-micromirror, as depicted in Figure 4(e), the total power collected exhibited a substantial increase of ∼2.87 times compared with the reference. The IR CCD image of the emitted beam was notably larger than the reference image. Despite the prediction of a 1.39-fold increase with an NA_obj_ of 0.65, as highlighted in Figure 2(j), the actual experimental results demonstrated further enhancement. This discrepancy can primarily be attributed to a slight reduction in the effective NA, as discussed in Figure S4(b). This decrease is mainly caused by an 8 μm thick spacer placed between the microdisk laser and the meta-micromirror. This setup results in distortions as the laser-emitted beam is reflected by the meta-micromirror and then recaptured by the objective lens. Additionally, the experimental setup’s various optical components might cause partial loss of highly diverged emitted beams, further decreasing the effective collection NA. Reverse analysis of the 2.87-fold enhancement noted in Supplementary Note 4 suggests that the effective NA_obj_ in our experiment was ∼0.56. The emission wavelengths of the microdisk laser placed on PDMS and the meta-micromirror were observed to be 1573.29 and 1575.5 nm, respectively, similar to the wavelength difference revealed in Figure 4(d), with the threshold powers measured at 1093 and 953 μW, respectively.

**Figure 5:**
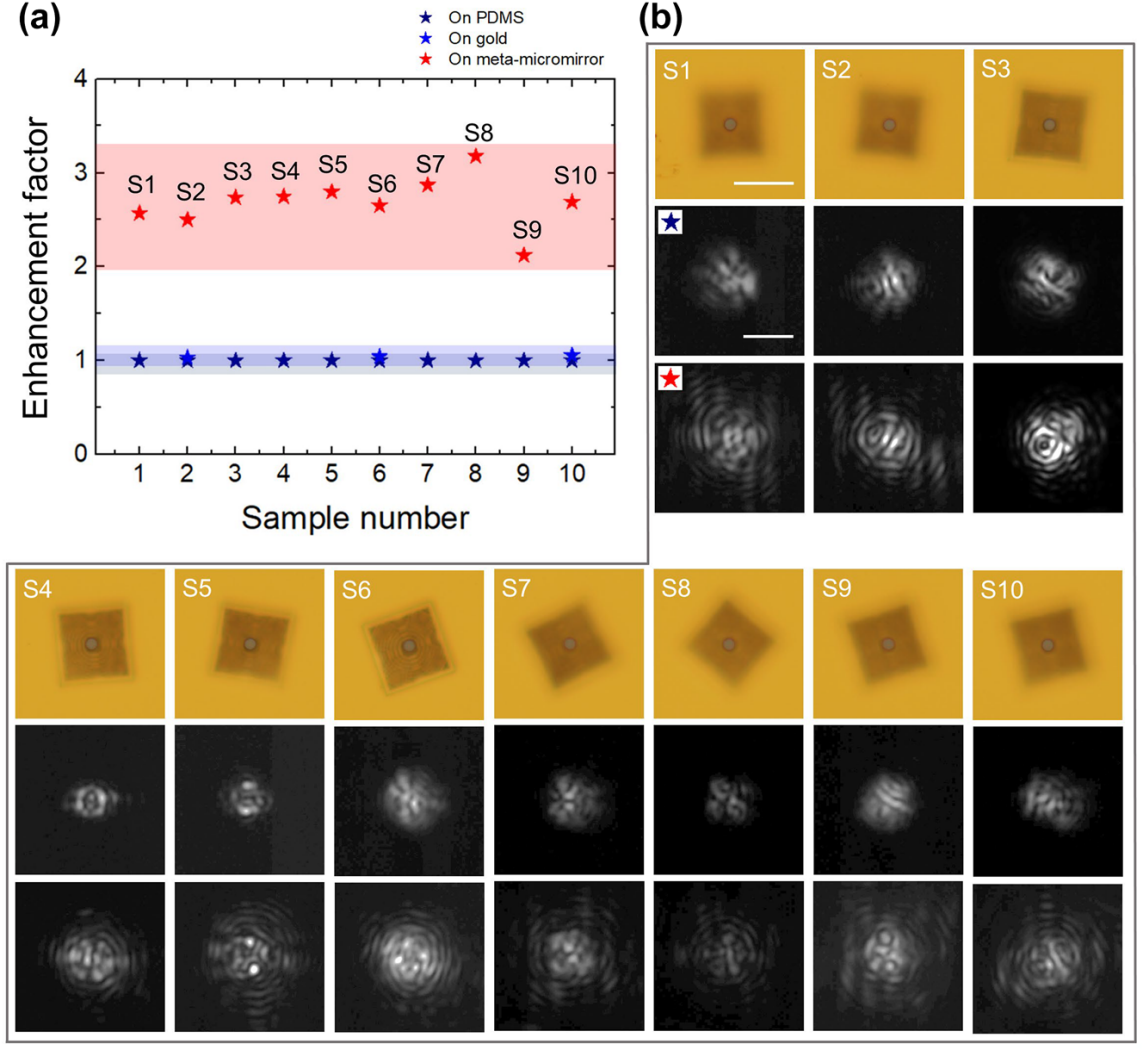
Statistical measurements with 10 samples. (a) Emission enhancement factors of 10 microdisk lasers integrated with meta-micromirrors (red stars) and enhancement factors of 3 microdisk lasers integrated with only gold reflectors (blue stars). (b) Microscopy images, IR CCD images (captured at a peak pump power of 1722 μW) of microdisk laser emission on PDMS, and IRCCD images of the same lasers integrated with meta-micromirrors, for the 10 samples in (a). The scale bars in the optical microscope image and IR CCD image represent 30 μm and 10 μm, respectively.

To enhance the accuracy of the performance validation, we assessed ten prepared samples by comparing them with the reference results and quantified the emission enhancement, as summarized in Figure 5(a), and the laser wavelengths and threshold powers are summarized in Supplementary Note 11. Additionally, the emission enhancement factors of the other three samples, which were mounted on Au-reflectors without metastructures, are overlaid in this graph corresponding to samples 2, 6, and 10, where the result in Figure 4(a) corresponds to sample 2. This figure clearly demonstrates that each sample equipped with the meta-micromirror exhibited a distinct emission enhancement, with the values ranging from a maximum of 3.18 to a minimum of 2.12. Variations in enhancement factors may result from a range of factors, including manufacturing process variations, alignment issues, measurement inaccuracies, and inconsistencies in the printing process. The average enhancement factor for these ten samples was 2.68. This significant enhancement is visually confirmed in Figure 5(b) by the IR CCD images, which distinctly demonstrate that all samples display considerably expanded emission images compared to their references [Supplementary Note 12]. For the three samples placed on the Au-reflector, the average enhancement factor was measured to be 1.04, reinforcing the conclusion that significant emission enhancement is not achievable in the structure with only an Au-reflector.

## 6 Conclusions

In this study, we demonstrated the emission engineering of a small laser to substantially enhance the collection efficiency through the integration of a high-NA meta-micromirror with a microdisk laser. This integration not only boosts the emission efficiency of microdisk lasers, but also facilitates meticulous control over their emission angles, establishing a formidable platform for advanced emission engineering. Our approach, utilizing reflective meta-micromirrors, is primarily focused on engineering downward emissions, which resulted in a remarkable 2.68-fold increase in the upward vertical emission from the microdisk laser. However, the potential of this platform may extend beyond its initial accomplishments. By amalgamating these reflective meta-micromirrors with transmissive metamaterials aimed at upward emission, we can surpass the current collection efficiency boundaries, thereby creating a more potent platform for emission control. Additionally, this integration has the potential to exploit a variety of metasurface applications, including orbital angular momentum manipulation, polarization control, beam steering, and holography, thereby providing comprehensive control over laser emissions. We are confident that our findings will contribute significantly to the advancement of small-laser technologies, paving the way for advanced applications in diverse areas such as photonic integrated circuits, biosensing technologies, the Internet of things (IoT), and the development of quantum light sources.

## Supplementary Material

Supplementary Material Details

## References

[1] Tucker R. S. (2011). Green optical communications-Part II: energy limitations in networks. *IEEE J. Sel. Top. Quantum Electron.*.

[2] Song D. I. (2022). Three-dimensional programming of nanolaser arrays through a single optical microfiber. *Optica*.

[3] Shih M. H., Hsu K. S., Lee K., Lai K. T., Lin C. T., Lee P. T. (2015). Compact tunable laser with InGaAsP photonic crystal nanorods for C-band communication. *IEEE J. Sel. Top. Quantum Electron.*.

[4] Wang X. (2023). Beam scanning and capture of micro laser communication terminal based on MEMS micromirrors. *Micromachines*.

[5] Yokoo A. (2017). Subwavelength nanowire lasers on a silicon photonic crystal operating at telecom wavelengths. *ACS Photonics*.

[6] Miao P. (2016). Orbital angular momentum microlaser. ..

[7] Noginov M. A. (2009). Demonstration of a spaser-based nanolaser. *Nature*.

[8] Miller D. A. B. (2009). Device requirements for optical interconnects to silicon chips. *Proc. IEEE*.

[9] Gao M., Wei C., Lin X., Liu Y., Hu F., Zhao Y. S. (2017). Controlled assembly of organic whispering-gallery-mode microlasers as highly sensitive chemical vapor sensors. *Chem. Commun.*.

[10] Kita S. (2008). Refractive index sensing utilizing a cw photonic crystal nanolaser and its array configuration. *Optica*.

[11] He L., Özdemir Ş. K., Zhu J., Kim W., Yang L. (2011). Detecting single viruses and nanoparticles using whispering gallery microlasers. *Nat. Nanotechnol.*.

[12] Hachuda S. (2013). Selective detection of sub-atto-molar Streptavidin in 10^13-fold impure sample using photonic crystal nanolaser sensors. *Opt. Express*.

[13] Choi J. H. (2016). A high-resolution strain-gauge nanolaser. *Nat. Commun.*.

[14] Kita S. (2011). Super-sensitivity in label-free protein sensing using a nanoslot nanolaser. *Opt. Express*.

[15] Feng J. (2019). Random organic nanolaser arrays for cryptographic primitives. *Adv. Mater.*.

[16] Fainman Y., Jiang S. (2021). Nanolasers: towards large-scale phase-locked laser arrays. *Technical Digest – International Electron Devices Meeting, IEDM*.

[17] Gather M. C., Yun S. H. (2011). Single-cell biological lasers. *Nat. Photonics*.

[18] Kim T.-il (2013). Injectable, cellular-scale optoelectronics with applications for wireless optogenetics. *Science*.

[19] Ma R. M., Oulton R. F. (2019). Applications of nanolasers. *Nat. Nanotechnol.*.

[20] Vahala K. J. (2003). Optical microcavities. ..

[21] Hill M. T., Gather M. C. (2014). Advances in small lasers. *Nat. Photonics*.

[22] Munnelly P. (2017). Electrically tunable single-photon source triggered by a monolithically integrated quantum dot microlaser. *ACS Photonics*.

[23] Jeong K. Y., Hwang M. S., Kim J., Park J. S., Lee J. M., Park H. G. (2020). Recent progress in nanolaser technology. *Adv. Mater.*.

[24] Smith D. R., Vier D. C., Koschny T., Soukoulis C. M. (2005). Electromagnetic parameter retrieval from inhomogeneous metamaterials. *Phys. Rev. E: Stat., Nonlinear, Soft Matter Phys.*.

[25] Landy N. I., Sajuyigbe S., Mock J. J., Smith D. R., Padilla W. J. (2008). Perfect metamaterial absorber. *Phys. Rev. Lett.*.

[26] Staude I., Schilling J. (2017). Metamaterial-inspired silicon nanophotonics. *Nat. Photonics*.

[27] Chen H. T., Taylor A. J., Yu N. (2016). A review of metasurfaces: physics and applications. *Rep. Prog. Phys.*.

[28] Holloway C. L., Kuester E. F., Gordon J. A., O’hara J., Booth J., Smith D. R. (2012). An overview of the theory and applications of metasurfaces: the two-dimensional equivalents of metamaterials. *IEEE Antennas Propag. Mag.*.

[29] Yu N. (2011). Light propagation with phase discontinuities: generalized laws of reflection and refraction. ..

[30] Balthasar Mueller J. P., Rubin N. A., Devlin R. C., Groever B., Capasso F. (2017). Metasurface polarization optics: independent phase control of arbitrary orthogonal states of polarization. *Phys. Rev. Lett.*.

[31] Arbabi A., Horie Y., Bagheri M., Faraon A. (2015). Dielectric metasurfaces for complete control of phase and polarization with subwavelength spatial resolution and high transmission. *Nat. Nanotechnol.*.

[32] Aieta F. (2012). Aberration-free ultrathin flat lenses and axicons at telecom wavelengths based on plasmonic metasurfaces. *Nano Lett.*.

[33] Lin Q. W., Wong H. (2018). A low-profile and wideband lens antenna based on high-refractive-index metasurface. *IEEE Trans. Antennas Propag.*.

[34] Kim J. Y. (2016). Highly tunable refractive index visible-light metasurface from block copolymer self-assembly. *Nat. Commun.*.

[35] Babicheva V. E., Evlyukhin A. B. (2021). Multipole lattice effects in high refractive index metasurfaces. *J. Appl. Phys.*.

[36] Fleury R., Sounas D. L., Alù A. (2014). Negative refraction and planar focusing based on parity-time symmetric metasurfaces. *Phys. Rev. Lett.*.

[37] Jaksic Z., Maksimovic M., Jakšić Z., Dalarsson N., Maksimović M. (2006). Negative refractive index metamaterials: principles and applications. ..

[38] Patel S. K., Sorathiya V., Lavadiya S., Luo Y., Nguyen T. K., Dhasarathan V. (2020). Numerical analysis of polarization-insensitive squared spiral-shaped graphene metasurface with negative refractive index. *Appl. Phys. B*.

[39] Jakšić Z., Vuković S., Matovic J., Tanasković D. (2010). Negative refractive index metasurfaces for enhanced biosensing. *Materials*.

[40] Liang H. (2018). Ultrahigh numerical aperture metalens at visible wavelengths. *Nano Lett.*.

[41] Fan Q., Liu M., Yang C., Yu L., Yan F., Xu T. (2018). A high numerical aperture, polarization-insensitive metalens for long-wavelength infrared imaging. *Appl. Phys. Lett.*.

[42] Kang M., Ra’Di Y., Farfan D., Alù A. (2020). Efficient focusing with large numerical aperture using a hybrid metalens. *Phys. Rev. Appl.*.

[43] Kotlyar V. V. (2017). Thin high numerical aperture metalens. *Opt. Express*.

[44] Zhang Q. (2020). High-numerical-aperture dielectric metalens for super-resolution focusing of oblique incident light. *Adv. Opt. Mater.*.

[45] Huang T. Y. (2019). A monolithic immersion metalens for imaging solid-state quantum emitters. *Nat. Commun.*.

[46] Park Y., Kim H., Lee J. Y., Ko W., Bae K., Cho K. S. (2020). Direction control of colloidal quantum dot emission using dielectric metasurfaces. *Nanophotonics*.

[47] Bao Y. (2020). On-demand spin-state manipulation of single-photon emission from quantum dot integrated with metasurface. ..

[48] Semnani B. (2023). Metasurface structures for control of quantum emitters. *SPIE – The International Society for Optical Engineering*.

[49] Dai W. (2020). Achieving circularly polarized surface emitting perovskite microlasers with all-dielectric metasurfaces. *ACS Nano*.

[50] Solntsev A. S., Agarwal G. S., Kivshar Y. Y. (2021). Metasurfaces for quantum photonics. *Nat. Photonics*.

