# Supplementary material for: Emission engineering in microdisk lasers via direct integration of meta-micromirrors
Source: Nanophotonics. 2024 Apr 17;13(16):2903–13. doi: 10.1515/nanoph-2023-0898 (PMC11501896; doi:10.1515/nanoph-2023-0898)
Supplement: Supplementary file 1 — Supplementary Material Details [file j_nanoph-2023-0898_suppl_001.pdf]

Supplementary Material

Emission engineering in microdisk lasers via direct integration of meta-micromirrors

Aran Yu[1], Moohyuk Kim[1], Da In Song[1,2], Byoung Jun Park[1], Hae Rin Jeong[1], Byeong Uk You[1], Seung-Woo Jeon[3], Sang-Wook Han[3,4], and Myung-Ki Kim[1,3,5]*

[1]*KU-KIST Graduate School of Converging Science and Technology, Korea University, Seoul, 02841, Republic of Korea*

[2]*Mechatronics Research Center, SAMSUNG Electronics, Hwaseong 18448, Republic of Korea*

[3]*Center for Quantum Information, Korea Institute of Science and Technology (KIST), Seoul, 02792, Republic of Korea*

[4]*Division of Nanoscience and Technology, KIST School, Korea University of Science and Technology (UST), Seoul, 02792, Republic of Korea*

[5]*Department of Integrative Energy Engineering, Korea University, Seoul 02841, Republic of Korea*

*rokmk@korea.ac.kr

Contents

1. Simulation of meta-micromirror
2. Scattering effects by meta-structures
3. Influence of resonant wavelength shift on efficiency
4. Influence of numerical aperture of objective lens and meta-micromirror on enhancement factor of collection efficiency
5. Fabrication of SiN meta-micromirror
6. Fabrication of InGaAsP microdisk laser
7. Experimental characterization of meta-micromirror
8. Influence of meta-micromirror on pumping efficiency
9. Investigation of PDMS residue
10. Experimental characterization of microdisk laser
11. Laser characteristics for 10 samples
12. Emission beam profile by meta-micromirror
13. Various practices to improve micro/nano laser emission

Supplementary Note 1: Simulation of meta-micromirror

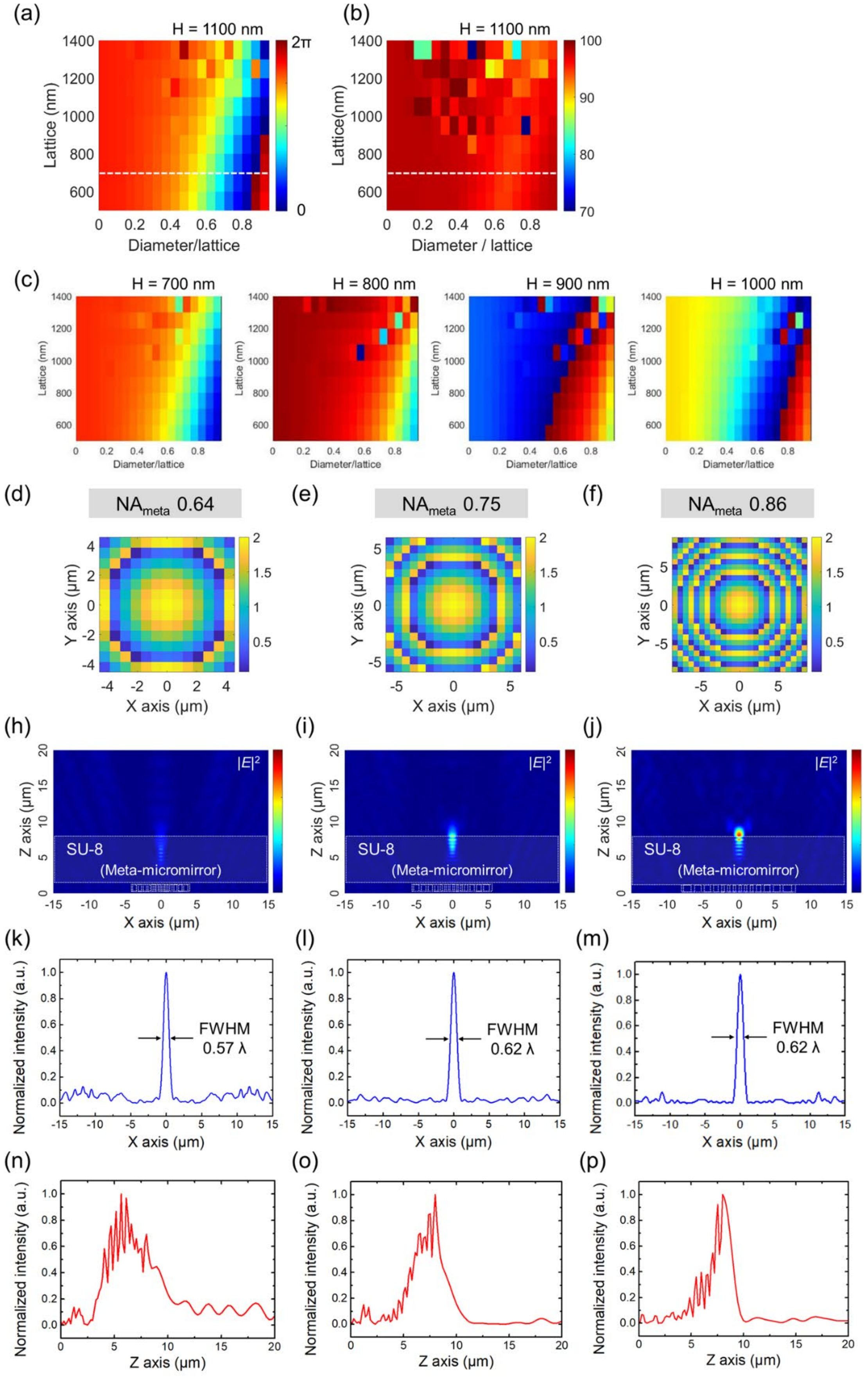

Fig. S1. (a-b) During the optimization of the unit cell, it is essential to consider both phase delay and reflectance simultaneously. The lattice exhibits resonance mode in the range from 800 nm to 1400 nm. Consequently, within this range, there are abrupt changes in phase delay and sudden drops in reflectance in certain intervals. When the lattice is above 1100 nm, the phase undergoes abrupt changes, making it challenging to achieve the desired phase even with small fabrication tolerances. Additionally, when the lattice is below 600 nm, it is challenging to implement a phase delay of 2π. Therefore, optimization was performed at 700 nm, and with increasing hole sizes, the average reflectance was maintained at 97% while achieving a 2π phase delay. Therefore, the lattice was optimized for 700 nm. As a result, with increasing hole size, a 2π phase delay was implemented, achieving a high average reflectance of 97%. (c) Phase delay of the unit cell as a function of hole size and lattice for heights ranging from 700 nm to 1000 nm. Compared to (a), it is evident that all cases fall short of achieving a 2π phase delay. (d-f) Phase maps designed to achieve meta-micromirrors with NA values of 0.64 (S = 9 μm), 0.75 (S = 12 μm), and 0.86 (S = 17 μm) when the meta-micromirror has a focal length of 8 μm (BG n = 1.61). (h-j) These are intensity xz field profile results from FDTD simulations illustrating the performance of the phase designs. They are normalized to Figure 2(e) as the reference. (h) The NA 0.64 meta-micromirror lacks the capability to engineer the emission from the microdisk laser with a large radiation angle effectively. Although light is focused, its efficiency is diminished. (i) The NA 0.75 meta-micromirror exhibits a similar behavior. (j) The NA 0.84 meta-micromirror demonstrates effective focusing. This implies that to accommodate the wide radiation angle of the microdisk laser with an NA of 0.87 or higher, a meta-micromirror with a matching NA is required. (k-m) These are x-axis profiles for meta-micromirrors at each NA. (k) The NA 0.64 meta-micromirror has a beam spot FWHM of 890.7 nm, which corresponds to 0.57λ. (l) The NA 0.75 meta-micromirror has a beam spot FWHM of 956.7 nm, equivalent to 0.62λ. (m) The NA 0.84 meta-micromirror exhibits a beam spot FWHM of 958.9 nm, which corresponds to 0.57λ. (n-p) These are z-axis profiles for meta-micromirrors at each NA. (n) The NA 0.64 meta-micromirror shows that the beam confinement is dominated by the Fabry-Pérot resonance effect within SU-8. (o) Although slightly improved, the NA 0.75 meta-micromirror exhibits a similar behavior. (p) The NA 0.84 meta-micromirror demonstrates pronounced beam confinement over the Fabry-Pérot resonance effect.

Supplementary Note 2: Scattering effects by meta-structures

To ensure that the observed efficiency improvement is due to the performance of the meta-micromirrors and not scattering, we undertook additional simulations, the results of which are presented in Fig. S2. Maintaining consistent conditions such as the total size and thickness, the SU-8 layer's thickness, and the lattice size, we altered the metasurface to function purely as a scatterer. This was achieved by setting all hole sizes to either 380 nm, representing an intermediate size of air holes in the metasurface, or to 480 nm, which corresponds to a π-phase shift. The far-field outcomes, illustrated in Fig. S2(a) and (b), indicate that there was no significant increase in the vertical emission component, with enhancement factors of 1.04 and 1.05 respectively, akin to those obtained using a gold reflector. Conversely, Fig. S2(c) demonstrates that incorporating a meta-micromirror with phase displacement significantly enhances the vertical emission component, thereby enhancing collection efficiency. Through the comparison of these scenarios, it is clear that the enhancement in efficiency is attributable to the meta-micromirrors' functionality, rather than to scattering effects.

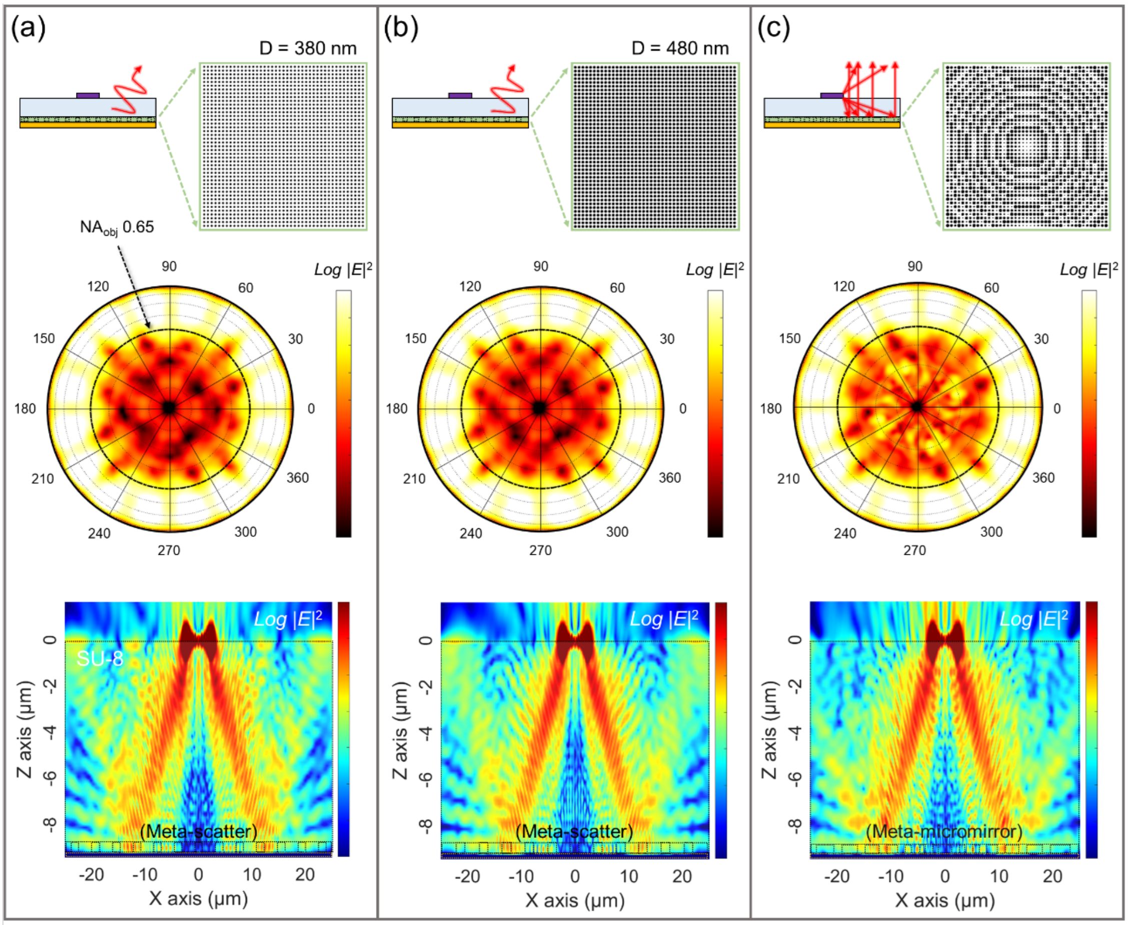

Fig. S2. Comparative Analysis of Meta-micromirror and Scattering Effects. (a) and (b) depict the far-field emission profiles for metasurfaces modified to act as simple scatterers with air hole sizes set at 380 nm and 480 nm, respectively, demonstrating no significant vertical emission enhancement with factors of 1.04 and 1.05, akin to using a gold reflector. (c) illustrates the enhanced vertical emission achieved through the introduction of a meta-micromirror with phase displacement, highlighting a substantial increase in collection efficiency. These comparisons affirm that the observed enhancement is attributable to the meta-micromirror's design and functionality, rather than mere scattering.

Supplementary Note 3: Influence of resonant wavelength shift on efficiency

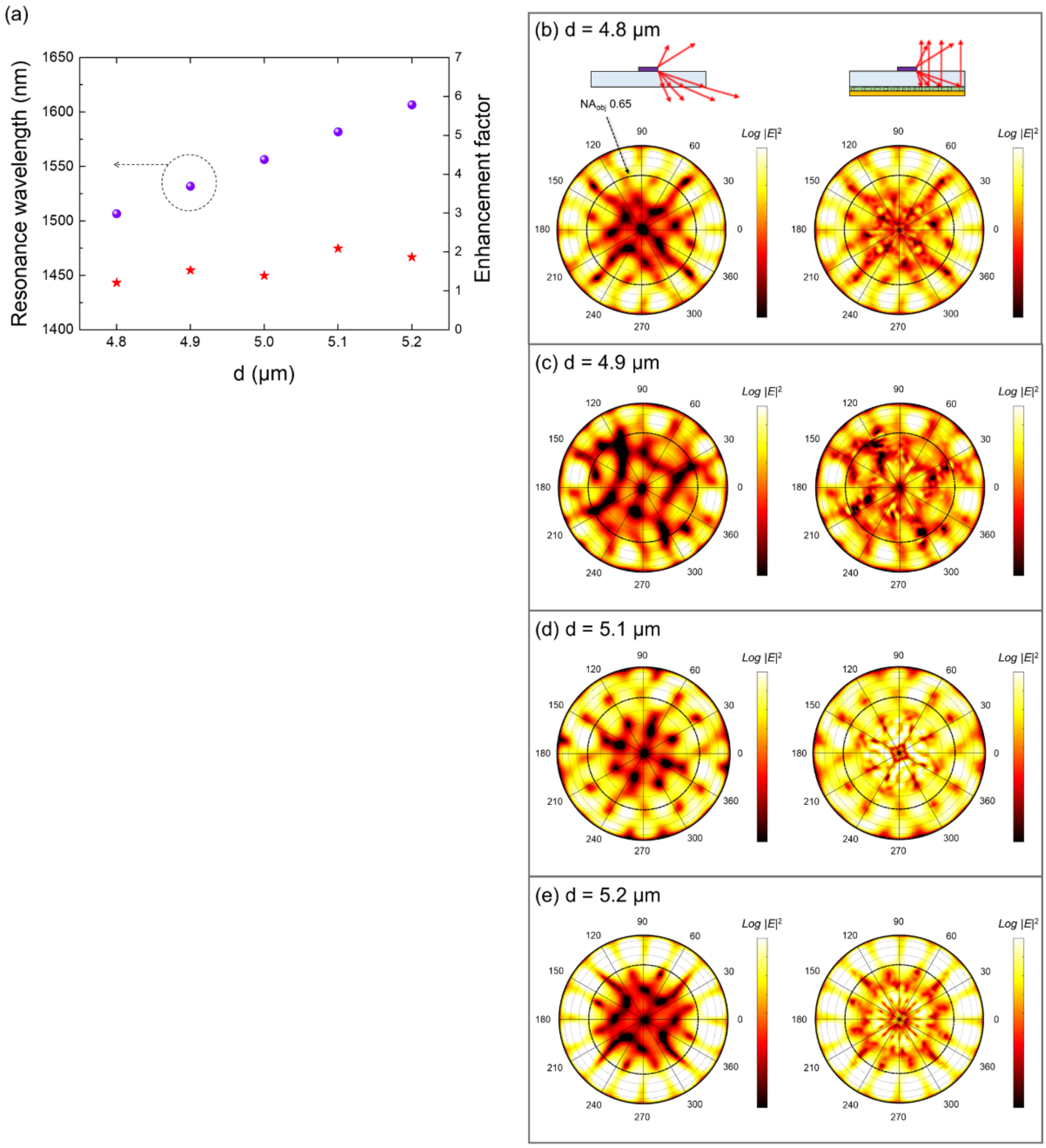

Fig. S3. Impact of microdisk diameter on emission enhancement with meta-micromirror. This figure showcases the results of full-field simulations designed to explore the effect of microdisk diameter variations on emission enhancement when coupled with a meta-micromirror optimized for 1550 nm. (a) illustrates that differences in the resonance wavelengths of microdisk lasers and emission enhancement factors are due to variations in disk diameters. Subsequent panels (b-e) detail the far-field simulation outcomes for microdisks of varying diameters: 4.8 μm (b), 4.9 μm (c), 5.1 μm (d), and 5.2 μm (e), with their respective resonance wavelengths of 1506.51 nm, 1531.74 nm, 1581.67 nm, and 1606.48 nm. The calculated enhancement factors for these diameters are 1.21, 1.53, 3.18, and 2.71, respectively, demonstrating that the meta-micromirror facilitates emission enhancement across a spectrum of wavelengths.

Supplementary Note 4: Influence of numerical aperture of objective lens and meta-micromirror on enhancement factor of collection efficiency

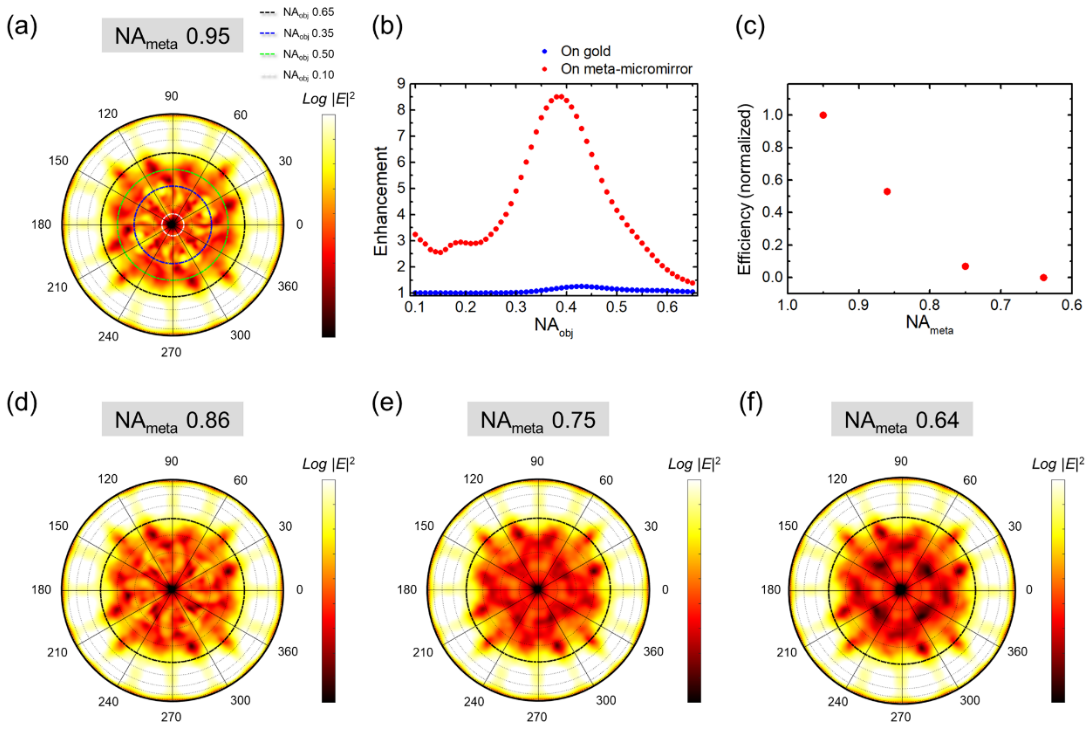

Fig. S4. (a) This illustration shows a saturated far-field distribution using a meta-micromirror with an NA_{meta} of 0.95. Objective lens NAs (NA_{obj}) are marked by thick dashed lines in different colors: black for 0.65 (40.5 degrees), green for 0.5 (30 degrees), blue for 0.35 (20.5 degrees), and white for 0.1 (5.7 degrees). (b) The graph details the enhancement factor of collection efficiency varying with NA_{obj}. (c) It also depicts the relative decrease in collection efficiency as NA_{meta} diminishes, normalized to the efficiency at NA_{meta} = 0.95. (d-f) Show far-field patterns for NA_{meta} values of 0.86, 0.75, and 0.64, respectively.

Supplementary Note 5: Fabrication of SiN meta-micromirror

In our metasurface design, we utilized effective index modulation by adjusting hole sizes to achieve phase changes and control wavefronts. This necessitated the use of silicon nitride (SiN) with consistent refractive indices and high transmittance levels. To attain these properties, we employed Chemical Vapor Deposition (CVD) for the deposition process. Nonetheless, direct SiN deposition on a gold (Au) substrate via plasma-enhanced chemical vapor deposition (PECVD) was impractical due to the high temperatures involved, which compromised the gold substrate by melting it, thereby hindering sample fabrication. Moreover, amorphous SiN, capable of being deposited at relatively lower temperatures, proved inadequate for our purposes due to its irregular refractive index and absorption issues. To overcome these obstacles, our approach involved depositing SiN to a thickness of 1100 nm on a silicon (Si) substrate using PECVD. We then patterned the metasurface with tethers and utilized transfer printing technology with a PDMS stamp to transfer the SiN metasurface onto the gold substrate. This technique enables us to preserve the SiN layer's uniformity and integrity, ensuring the metasurface's performance is not compromised by the limitations associated with direct deposition on gold.

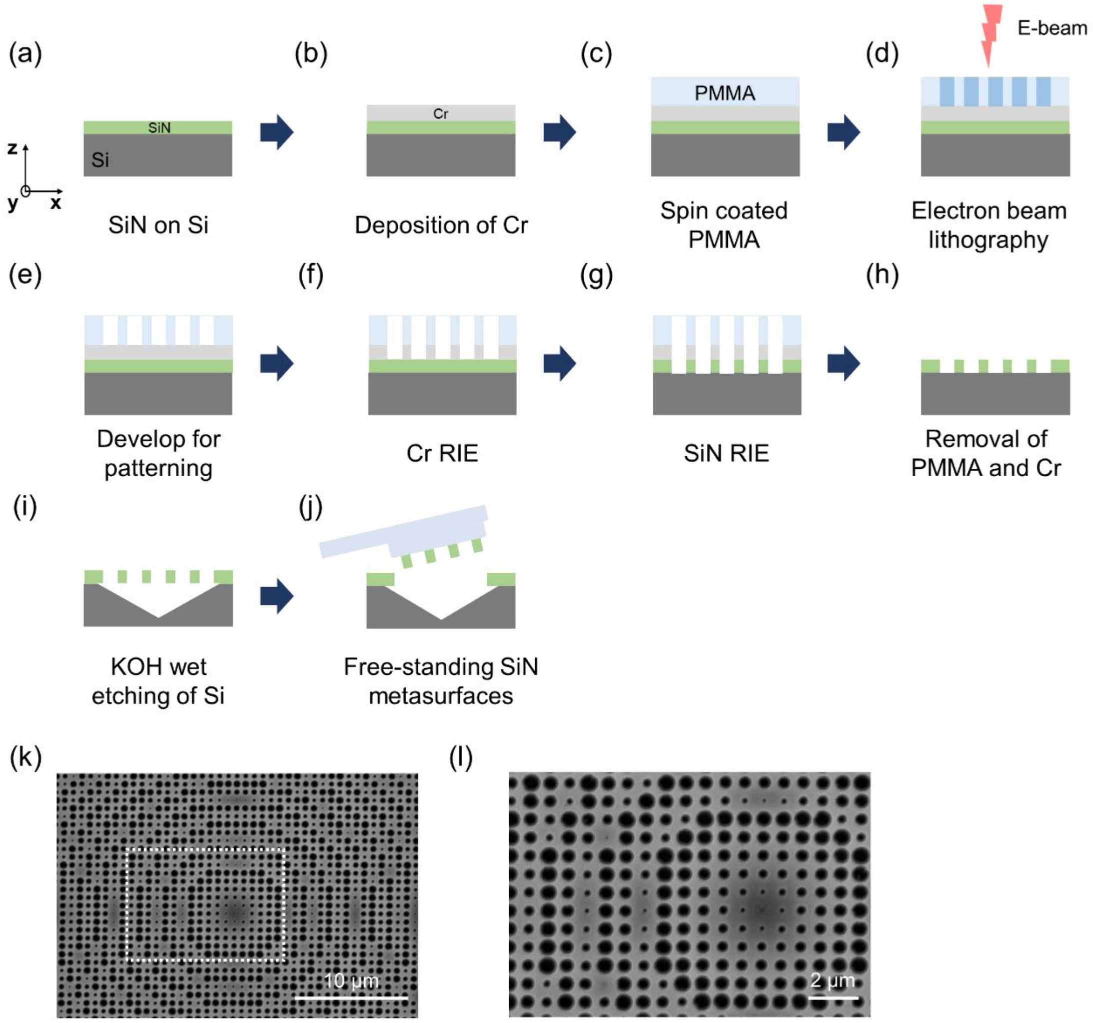

Fig. S5. The process for creating free-standing structures on a silicon nitride metasurface involves several steps. (a) Initially, a silicon nitride layer, nominally 1100 nm thick, is deposited on silicon. The process has an approximate error margin of 100 nm, resulting in an actual thickness of about 1040 nm in our experiments. (b) Next, a 50 nm-thick chromium (Cr) layer is applied as a hard mask through e-beam evaporation. (c) For e-beam lithography preparation, PMMA 950 C4 resist is spin-coated at 2000 rpm for 45 seconds and pre-baked at 180˚C

for 80 seconds, achieving a thickness of roughly 450-500 nm. (d) E-beam lithography, following the hole map in Figure 2(d), patterns tethers measuring 2 μm in length and 500 nm in width at each edge to create free-standing structures. Patterning a set of four 30 μm metasurfaces typically takes approximately 5 minutes, using a dose condition of 380 $\mu C/cm^2$ and an acceleration voltage of 30 kV. (e) The development process involves a 90-second treatment in MIBK:IPA at a 1:3 ratio, followed by an IPA rinse and a 90-second post-bake at 90˚C. (f) Chromium (Cr) RIE (Reactive Ion Etching) uses PMMA as the mask. (g) SiNx RIE is then performed, using the Cr layer as the etching mask. (h) PMMA removal involves soaking in acetone on a hot plate at 40°C for 20 minutes and an O_2 plasma treatment at 100 watts for 10 minutes. Any residual hardened PMMA debris is cleared with a Piranha treatment, followed by a 2-minute Cr etching step to remove the Cr layer. (i) Potassium Hydroxide (KOH) etching at 80˚C for 40 minutes etches away the silicon substrate under the silicon nitride. (j) The tethers are then severed using mechanical force, applied via a PDMS stamp with the assistance of a nano-stage. (k-l) The SEM images display the metasurface with holes drilled as per the hole map. The variation between the intended and the actual sizes of the air holes ranges from a minimum of ±0.5% to a maximum of ±5.3%

Supplementary Note 6: Fabrication of InGaAsP microdisk laser

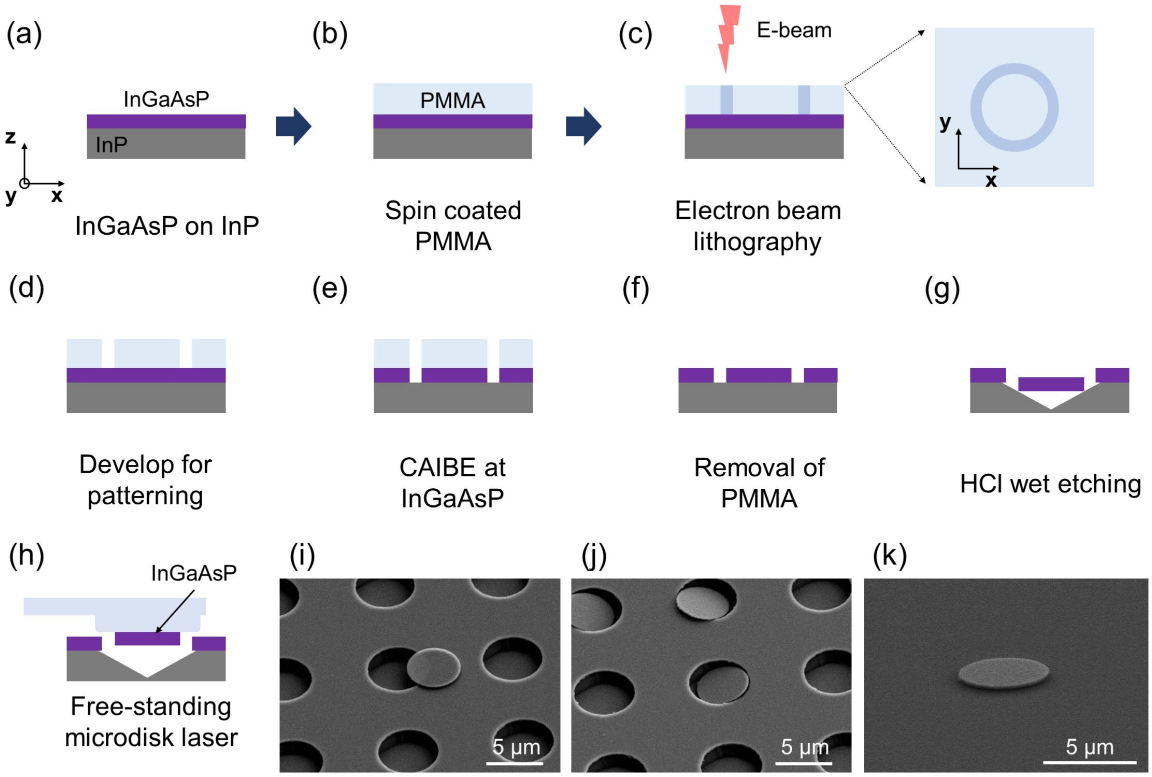

Fig. S6. The process for fabricating transferable InGaAsP microdisk lasers, includes several steps. (a) It begins with an InGaAsP layer on an InP substrate. (b) PMMA 950 C4 is then spin-coated using e-beam resist at 4000 rpm for 45 seconds, followed by pre-baking at 180°C for 80 seconds, resulting in a PMMA layer approximately 350-400 nm thick. (c) E-beam lithography is employed to create a 5 μm-diameter disk in a donut shape. (d) The developed structure is then treated in MIBK: IPA (1:3) for 90 seconds, rinsed in IPA, and post-baked at 90°C for another 90 seconds. (e) The InGaAsP is etched using Chemical Assisted Ion Beam Etching (CAIBE). (f) PMMA removal involves soaking the structure in acetone on a hot plate at 40°C for 20 minutes, followed by an O_2 plasma treatment at 100 watts for 10 minutes. (g) A critical wet etching step is then conducted in an HCl: H_2O solution (3:1 ratio) for 1 minute. This step requires precise timing and optimization, monitored under an optical microscope at 10-second intervals, until the microdisk laser slightly settles. (h) The slight settling of the microdisk laser facilitates its transfer onto a PDMS stamp. (i-k) SEM images illustrate the InGaAsP microdisk laser post-wet etching: (i) showing the partially exposed microdisk laser still attached to the substrate, (j) depicting the microdisk laser suspended across a gap, and (k) featuring a microdisk laser printed on SU-8 near the focus spot of the meta-micromirror.

Supplementary Note 7: Experimental characterization of meta-micromirror

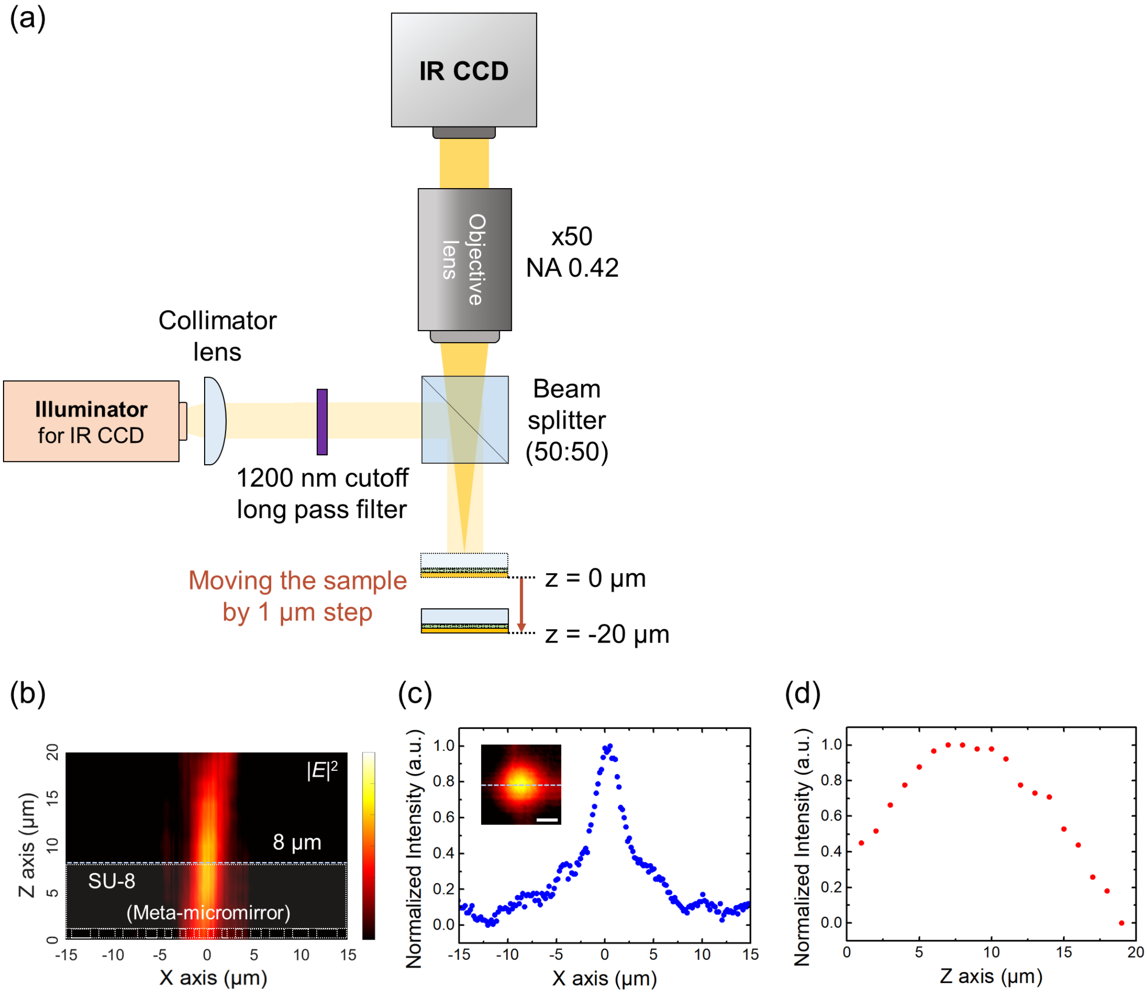

Fig. S7. (a) The experimental setup for characterizing the meta-micromirror is depicted in a schematic diagram. The setup includes an illuminator covering a wavelength range of 400 - 1750 nm, with collimation achieved using a plano-convex lens. A long-pass filter is used to filter out wavelengths above 1200 nm. The light then passes through a 50:50 beam splitter and is directed onto the sample. The sample stage is meticulously lowered by 1 μm increments to scan the z-axis of the meta-micromirror. The light, once reflected and focused by the meta-micromirror, travels through an IR long-focal lens (x50, NA 0.42) before being captured by an IR CCD camera. (b) The xz field profile of the meta-micromirror, as shown, illustrates the focal point near the end of the SU-8, closely matching the simulation results seen in Figure 2(e). (c) These profiles show the Gaussian-shaped beam spot at the focal point (with a 2 μm scale bar) and the x-axis profile. Fitting this profile with a Gaussian function reveals a half-width of 3.88 μm. The beam's reflectance efficiency near the focus is measured at 46.78%. The larger half-width, as compared to the simulation, is attributed to the use of an illuminator with a wavelength range of 1200 nm to 1750 nm, and potentially due to the limiting numerical aperture (NA) of the objective lens (0.42). (d) The z-axis profile demonstrates a depth of field (DoF) of 10.68 μm.

Supplementary Note 8: Influence of meta-micromirror on pumping efficiency

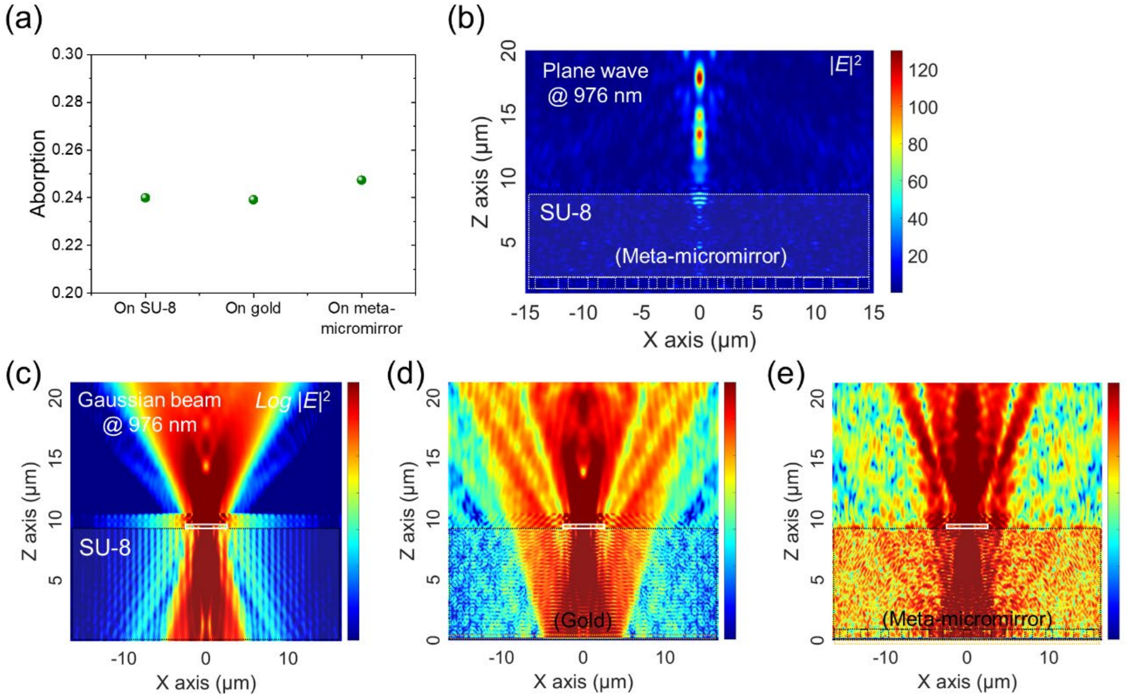

Fig. S8. Simulation results for meta-micromirror performance at 976 nm pumping wavelength. (a) shows the absorption power in the microdisk laser for three different structures: SU-8 only, SU-8/gold, and SU-8/meta-micromirror. The meta-micromirror exhibits a slight increase in absorption compared to SU-8 and gold, but the difference is negligible. (b) illustrates the meta-micromirror's performance at 976 nm, presenting an xz intensity profile of a parallel beam that reflects off the meta-micromirror and focuses at a distance of 17.8 μm, indicating a longer focal length and a reduced numerical aperture compared to a wavelength of 1550 nm. Panels (c-e) display xz intensity log profiles highlighting the effects of structure changes on the microdisk's absorption when exposed to a 976-nm Gaussian beam. (c) depicts the absorption by the microdisk laser on the surface of the SU-8, (d) illustrates the beam reflection from a gold surface, maintaining its incident angle, and (e) shows how the beam at 976 nm wavelength is focused and converged upward by the meta-micromirror, resulting in a slight increase in the microdisk's absorption.

Supplementary Note 9: Investigation of PDMS residue

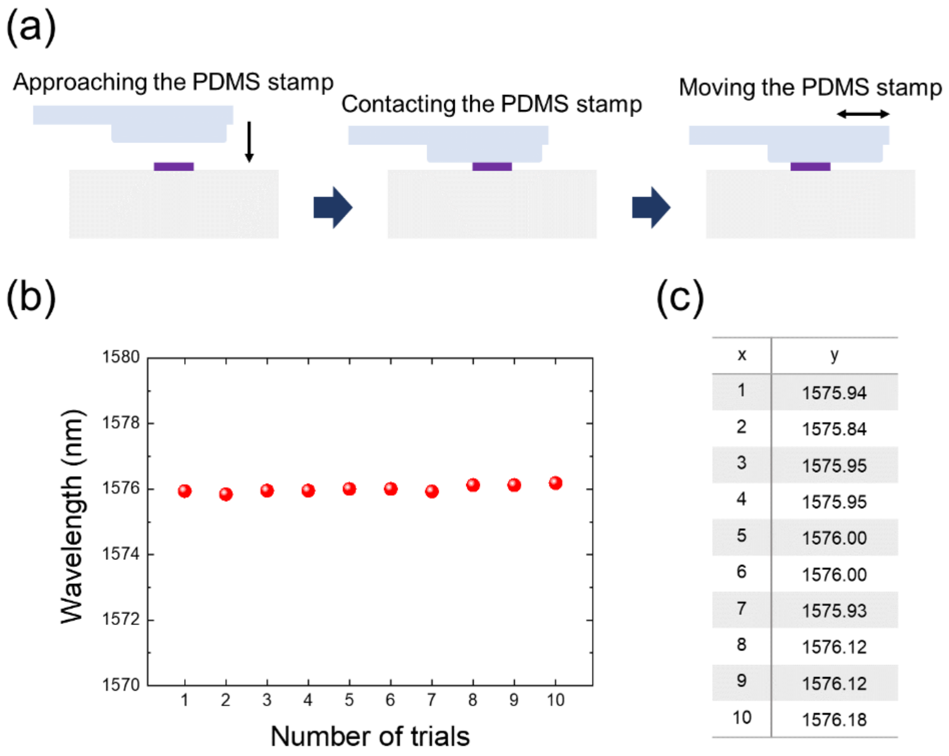

Fig. S9. Investigation of PDMS residue impact on microdisk laser. (a) Schematic representation of the experimental setup used to assess the effect of PDMS stamp residue on microdisk lasers. The procedure involved attaching the PDMS stamp to the microdisk, ensuring thorough contact by movement, then detaching it, with each attach-detach cycle constituting one trial. (b) shows the laser wavelength variations across different trials, employing the methodology depicted in (a). (c) displays the detailed data obtained from these trials, highlighting a maximum wavelength shift of 0.18 nm around an average wavelength of 1576 nm. This variation is considered within the bounds of experimental error, thereby confirming that PDMS residues have a negligible impact on the transfer printing process.

Supplementary Note 10: Experimental characterization of microdisk laser

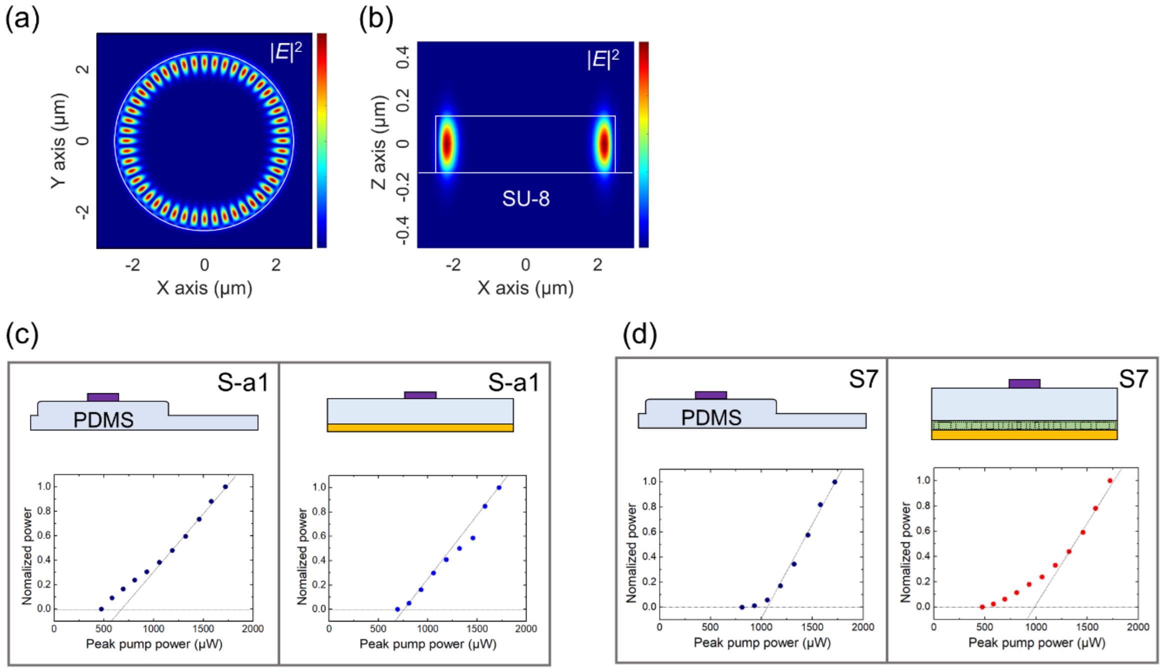

Fig. S10. (a-b) The FDTD simulation presents intensity profiles in the xy and xz planes of a microdisk laser situated on SU-8. This result exhibits a resonance wavelength of 1556.23 nm and an azimuthal mode number (M) of 24. When the microdisk is placed on PDMS (with a refractive index n = 1.395), there is a noticeable shift in the resonance wavelength to 1549.84 nm. (c-d) These panels correlate with Fig. 4(d-e) and display the output powers as a function of peak pump power. (c) In the left panel, the microdisk laser on PDMS showcases a threshold power of 674 μW, while the right panel, featuring the microdisk laser on the gold reflector, shows a threshold power of 772 μW. (d) The left panel depicts the microdisk on PDMS, with a threshold power of 1093 μW. The right panel illustrates the microdisk in proximity to the focal point of the meta-micromirror, demonstrating a threshold power of 953 μW.

Supplementary Note 11: Laser characteristics for 10 samples

As demonstrated in Fig. S11(a), a systematic shift in wavelength occurs when the microdisk laser is positioned on the meta-micromirror. This shift is attributed to the refractive index difference between the PDMS (n = 1.395) and the SU-8 spacer used on the meta-micromirror (n = 1.61). Our simulations indicate that this refractive index disparity can lead to a wavelength difference of up to 6.39 nm for the same lasing mode. However, the actual shift observed can vary due to several experimental factors, such as the refractive index variability of PDMS and SU-8, and the microdisk's contact state after transfer printing. In Fig. S11(a), the observed shifts range from a minimum of 0.96 nm (S10 sample) to a maximum of 5.92 nm (S9 sample), highlighting the influence of these conditions on the wavelength displacement.

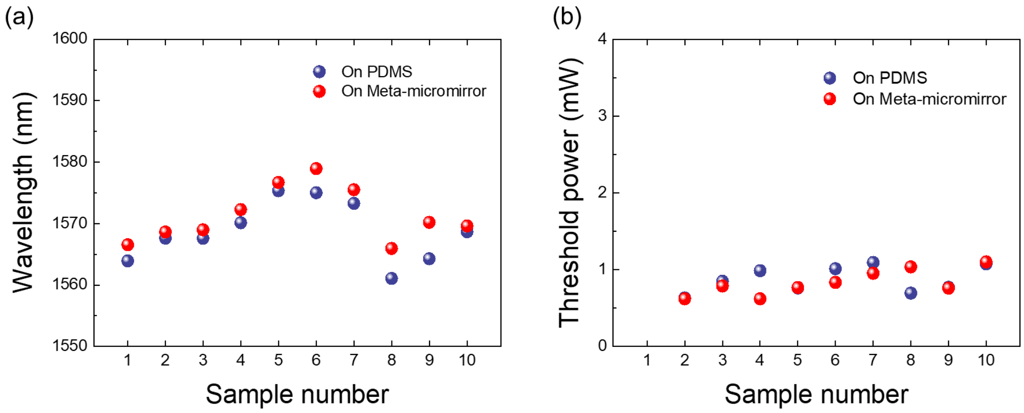

Fig. S11. (a) Laser wavelengths, and (b) threshold powers for 10 samples as referenced in Fig. 5. Samples mounted on PDMS (shown in dark blue) have an average wavelength of 1568.69 nm and a mean threshold power of 847.8 μW. In contrast, samples integrated with the meta-micromirror (depicted in red) feature an average wavelength of 1571.33 nm, with a mean threshold power of 831.12 μW. It is noted that the threshold power for sample 1 was not recorded.

Supplementary Note 12: Emission beam profile by meta-micromirror

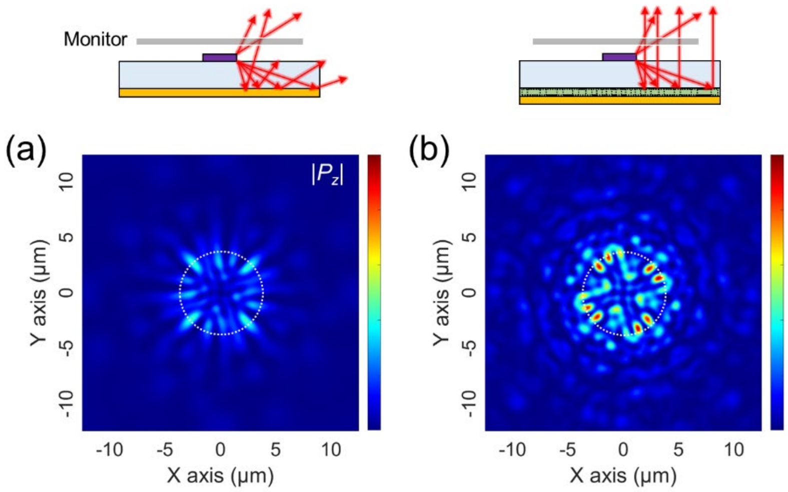

Fig. S12. Comparative analysis of emission beam collimation by meta-micromirror. This figure illustrates the notable difference in emission beam size due to the collimation effect of a meta-micromirror. (a) displays the emission profile of a microdisk laser with a gold reflector, monitored 1.5 μm above the emission source. (b) reveals the emission profile with the meta-micromirror. This contrast underscores the meta-micromirror's ability to enhance beam size and collimation, correlating with the experimentally observed CCD images shown in Fig. 5, and providing a clear depiction of the vertical emission beam's expansion.

Supplementary Note 13: Various practices to improve micro/nano laser emission

Method		Performance enhancement
Geometric modification	Introducing Slits [1]	Directional Emission
	Introducing Grating [2]	Efficiency Enhancement, Directional Emission
	Using Tailoring Exceptional Points [3]	Efficiency Enhancement
	Using Notched Cavities [4]	Directional emission
	Improving Design of Photonic Crystals [5, 6]	Efficiency Enhancement, Directional Emission
	Introducing Imprinting Nanograting [7]	Efficiency Enhancement, Directional Emission
Reflector introduction	Introducing Mirror [8]	Efficiency Enhancement, Directional Emission
	Introducing DBR [9]	Efficiency Enhancement
Metasurface introduction	Introducing Metalens [10]	Directional Emission
Meta-mirror introduction	Introducing Meta-micromirror (★this work)	Efficiency Enhancement Directional Emission

Table S1. Overview of various strategies for enhancing micro/nano laser emission. This table presents a curated list of various approaches aimed at improving laser emission efficiency, including traditional geometries like reflectors, slits, gratings, and innovative designs such as photonic crystals and distributed Bragg reflectors (DBRs). Due to the distinct light collection mechanisms and objectives inherent to each method, a straightforward comparison with our approach is not feasible. For a comprehensive understanding, each strategy is accompanied by references, offering a broad perspective on the current advancements in laser emission enhancement techniques.

[1] G. Zhu et al., "Whispering-Gallery Mode Lasing in a Floating GaN Microdisk with a Vertical Slit," Sci Rep **10**(1), Nature Research (2020) [doi:10.1038/s41598-019-57118-y].

[2] L. Mahler et al., "Vertically emitting microdisk lasers," Nat Photonics **3**(1), 46–49 (2009) [doi:10.1038/nphoton.2008.248].

[3] Z. Zhang et al., "Elimination of Spatial Hole Burning in Microlasers for Stability and Efficiency Enhancement," ACS Photonics **5**(8), 3016–3022, American Chemical Society (2018) [doi:10.1021/acsphotonics.8b00800].

[4] Q. J. Wang et al., "Whispering-gallery mode resonators for highly unidirectional laser action" [doi:10.1073/pnas.1015386107/-/DCSupplemental].

[5] M. Narimatsu et al., "Enhancement of vertical emission in photonic crystal nanolasers," Appl Phys Lett **100**(12) (2012) [doi:10.1063/1.3696056].

[6] Y. Wang et al., "Improvement of Optical Confinement for Terahertz Vertical-Cavity Surface-Emitting Laser with Square-Lattice Photonic Crystal Structure," Coatings **13**(6), MDPI (2023) [doi:10.3390/coatings13060972].

[7] A. Y. Zhizhchenko et al., "Directional Lasing from Nanopatterned Halide Perovskite Nanowire," Nano Lett **21**(23), 10019–10025, American Chemical Society (2021) [doi:10.1021/acs.nanolett.1c03656].

[8] S.-H. Kim, J. Huang, and A. Scherer, "From vertical-cavities to hybrid metal/photonic-crystal nanocavities: towards high-efficiency nanolasers" (2012).

[9] K. Ishizaki et al., "Enhancement of slope efficiency of a dually modulated photonic-crystal surface-emitting laser over a wide range of emission angles by introducing a backside reflector," Journal of the Optical Society of America B **40**(2), 326, Optica Publishing Group (2023) [doi:10.1364/josab.472869].

[10] Y. Y. Xie et al., "Metasurface-integrated vertical cavity surface-emitting lasers for programmable directional lasing emissions," Nat Nanotechnol **15**(2), 125–130, Nature Research (2020) [doi:10.1038/s41565-019-0611-y].
